# Impact of In-Process Crystallinity of Biodegradable Scaffolds Fabricated by Material Extrusion on the Micro- and Nanosurface Topography, Viability, Proliferation, and Differentiation of Human Mesenchymal Stromal Cells

**DOI:** 10.3390/polym15061468

**Published:** 2023-03-15

**Authors:** Ognjan Lužanin, Vera Gudurić, Anne Bernhardt, Dejan Movrin, Ljiljana Damjanović-Vasilić, Pal Terek, Gordana Ostojić, Stevan Stankovski

**Affiliations:** 1 Faculty of Technical Sciences, University of Novi Sad, 21000 Novi Sad, Serbia; 2 Centre for Translational Bone, Joint and Soft Tissue Research, Faculty of Medicine Carl Gustav Carus, Technical University Dresden, 01307 Dresden, Germany; 3 Faculty of Physical Chemistry, University of Belgrade, 11000 Belgrade, Serbia

**Keywords:** material extrusion, human mesenchymal stromal cells, polymer crystallinity, nanosurface topography, cell response

## Abstract

Due to affordability, and the ability to parametrically control the vital processing parameters, material extrusion is a widely accepted technology in tissue engineering. Material extrusion offers sufficient control over pore size, geometry, and spatial distribution, and can also yield different levels of in-process crystallinity in the resulting matrix. In this study, an empirical model based on four process parameters—extruder temperature, extrusion speed, layer thickness, and build plate temperature—was used to control the level of in-process crystallinity of polylactic acid (PLA) scaffolds. Two sets of scaffolds were fabricated, with low- and high-crystallinity content, and subsequently seeded with human mesenchymal stromal cells (hMSC). The biochemical activity of hMSC cells was tested by examining the DNA content, lactate dehydrogenase (LDH) activity, and alkaline phosphatase (ALP) tests. The results of this 21-day in vitro experiment showed that high level crystallinity scaffolds performed significantly better in terms of cell response. Follow-up tests revealed that the two types of scaffolds were equivalent in terms of hydrophobicity, and module of elasticity. However, detailed examination of their micro- and nanosurface topographic features revealed that the higher crystallinity scaffolds featured pronounced nonuniformity and a larger number of summits per sampling area, which was the main contributor to a significantly better cell response.

## 1. Introduction

Tissue engineering (TE) is one of the engineering and scientific areas which have successfully utilized the intrinsic ability of additive manufacturing (AM) to provide control over the architecture of the constructs while providing standardization and reproducibility of the fabrication process. Among the commercially available AM technologies, selective laser sintering (SLS), binder jetting (3DP), fused deposition modeling (FDM), and fused filament fabrication (FFF) have proved to be among the most suitable technologies for the fabrication of such porous structures [1]. In addition to being affordable, FFF offers a high degree of precision in the X-Y direction, the versatility of material deposition patterns, and the satisfactory structural integrity of scaffolds [2]. Due to the continuous expansion of the spectrum of biofabrication technologies, FFF continues to progress, and certain material filaments such as polylactic acid and other polyesters are being certified as medical-grade materials [3]. Another important feature of FFF technology is the ability to control the in-process crystallinity of fabricated parts, i.e., the crystallinity content, which is solely the result of material extrusion, without any postprocessing. Several recent investigations indicate that in-process crystallinity can be controlled by an appropriate selection of FFF processing parameters [4,5,6,7,8,9,10]. This feature can be exploited to advantage in tissue engineering since various studies have shown that polymer crystallinity significantly affects scaffold cell response. In one of the early works on this topic, Park and Cima [11] demonstrated the faster spheroid formation of hepatocytes and lower fibroblast growth on PLLA crystalline samples. Opposed behavior of the two cell types depending on the PLA crystallinity was also shown by Sarasua et al. [12] with contrasting proliferation rates of fibroblasts and keratinocytes on samples with different crystalline structures. The same cell response was observed by Cui and Sinko [13], who showed improved fibroblast growth on crystalline scaffolds, while the osteoblasts preferred amorphous surfaces. Washburn et al. [14] fabricated gradients of polymer crystallinity on films of poly(L-lactic acid) by applying gradient annealing temperature. The resulting variations in crystallinity lead to changes in surface roughness on nanometer length scales, allowing the authors to demonstrate a lower proliferation rate of MC3T3-E1 osteoblasts on the more crystalline samples. Salgado et al. [15] investigated how crystallinity and the molecular weight of PLLA influence the behavior of the human osteosarcoma cell line (SaOS-2) and found that cells showed more spread morphology and higher adhesion on crystalline samples, while their proliferation rate was higher on amorphous surfaces. An improved morphology of cells on samples of higher crystallinity was also shown by Qiangying et al. [16] who used rat bone marrow stromal cells. This was followed by increased cell proliferation, differentiation, and total protein amount, showing improved cell–material interaction. Taken together, this body of work suggests that by controlling the crystallinity level, depending on the cell type, it is possible to guide cell responses in tissue engineering.

In addition to crystallinity, the nanoscale topography of scaffolds is another important factor that provides a crucial signaling modality in controlling cell function. With this in mind, various studies have focused on creating a synthetical nanoscale topography of scaffolds and substrates, with the primary goal being to improve control of cell attachment and proliferation. Experimenting with engineered nanoscale topography gradients, Goreham et al. [17] demonstrated that there is a specific nanotopography scale that encourages cell adhesion and spreading. De Peppo et al. [18] engineered titanium-coated hemispherical topographic nanostructures of different sizes, to control the morphology, proliferation, and osteogenic differentiation of human mesenchymal stem cells (hMSC) using colloidal lithography in combination with coating technologies. Onesto et al. [19] examined the interaction between neural cell networks and cell substrates, using nanotopography controlled by wet etching of rough silicon substrates. They reported that surface roughness significantly affected network topology. Employing plasma-based techniques to generate culture substrates, Sarasua et al. [12] controlled their nanotopographical features in combination with three differently tailored surface chemical functionalities. They showed that nanotopography influenced cell adhesion, spreading, and proliferation, while chemistry affected cell renewal and differentiation. Using nanosecond laser structuring, Veiko et al. [20] modified the chemical composition and surface relief of titanium discs used for seeding hMSC. They developed three types of surface reliefs with different spatial periods and found that the structures with subcellular to cellular periods were most beneficial for the cells’ life-sustaining activities. Malinauskas et al. [21] used fused filament fabrication to create biodegradable PLA scaffolds with appropriate porosity for cell seeding. Using direct laser writing ablation, the surfaces of scaffolds were treated to modify nanoscale surface roughness and increase connection with the cells. Jaidev and Chatterjee [22] conducted experiments using 3D-printed plain and modified PLA scaffolds with 70% porosity. The modification consisted of a polyethyleneimine and citric acid graft, after which calcium phosphate was deposited. They reported that the surface modification contributed to an increase of 50% in the adhesion and proliferation of mesenchymal stem cells. A study on 3D-printed PLA scaffolds [23] has reported that the chemical modification by etching in an alcohol–alkali solution improved osteogenesis in comparison with the untreated PLA scaffolds. In a recent report, Luo et al. [24] systematized synthetical nanotopographies for promoting cell differentiation based on their features and arrangement. In addition to previously discussed methods of topographical modifications, alternative methods of surface modifications were used to improve the scaffolds’ biomimetic properties for tissue engineering. According to the literature, the surfaces of scaffolds based on aliphatic polyesters have been modified using a wide range of methods, from mineral deposition and protein adsorption to functionalization and biomacromolecule cross linking and entrapment [25].

Based on the presented review of the literature, it has been well established that attachment, viability, proliferation, and differentiation of various cell types are impacted by their interaction with their physical, mechanical, and biological nanoscale environment. The 3D-printed scaffolds are therefore expected to provide numerous features which are central to the generation of the biochemical and physical signals for the cells. The reviewed studies also imply that the crystallinity of the polymer scaffold matrix is one of the crucial factors affecting this requirement, while, on the other side, we have also seen reports of successful control of in-process polymer crystallinity using FFF 3D printing. This brings to light the assumption that the biomimetic properties of 3D-printed scaffolds could be significantly impacted solely by controlling the in-process crystallinity of the resulting matrix. With this in mind, the basic idea behind this study is to utilize the FFF extrusion to fabricate scaffolds with targeted low- and high-level crystallinity, and conduct cell viability, proliferation, and differentiation analyses to establish which level of crystallinity is preferable in terms of cell attachment, growth, and differentiation. Subsequent testing of micro- and nanosurface topography, contact angle, and module of elasticity will be used to examine the differences generated by the choice of processing parameters, which will allow identification of the most important contributors to cell response within the two types of 3D-printed scaffolds.

## 2. Materials and Methods

### 2.1. Selection of Processing Parameters

**Figure 1:**
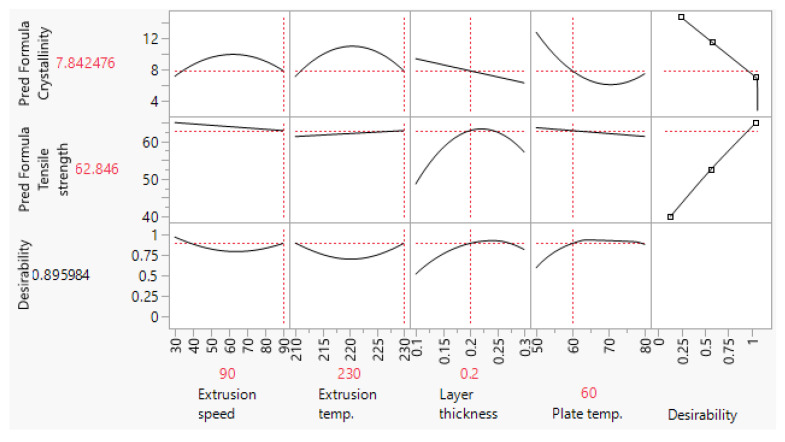
Optimization profiler showing FFF parameter settings which allow fabrication of scaffolds with low-level crystallinity (LLC) and high-tensile strength.

**Figure 2:**
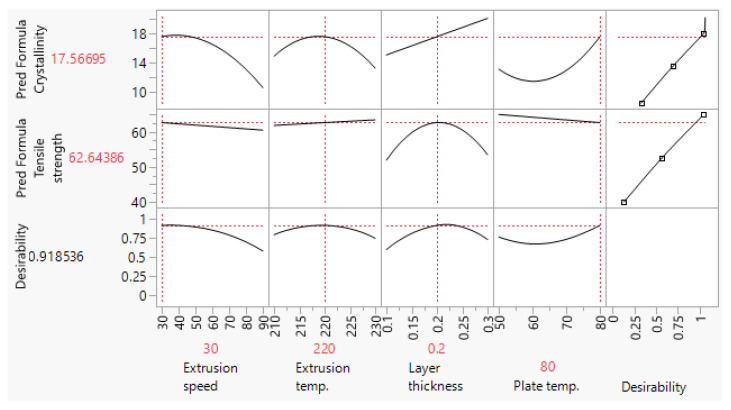
Optimization profiler showing FFF parameter settings which allow fabrication of scaffolds with high-level crystallinity (HLC) and high tensile strength.

The FFF processing parameter values used for the fabrication of scaffolds are based on the methodology presented in [9]. In a designed experiment, extrusion speed (ES), extruder temperature (ET), layer thickness (LT), and build plate temperature (PT) were systematically varied to generate two regression models which allowed independent predictions of the in-process crystallinity content of the specimens, Equation (1), and their tensile strength, Equation (2). In this study, these two prediction expressions were used to perform dual optimization and generate two sets of parameters (Figure 1 and Figure 2) which yield scaffolds with low- and high-level crystallinity, while maintaining tensile strength at a high level. The settings shown in the two optimization profilers (Figure 1 and Figure 2), were subsequently used to fabricate these two types of scaffolds. Statistical analyses and dual optimization were performed in JMP r14 (SAS, Inc., Cary, NC, USA).

(1) $$\begin{array}{c} {\hat{Y}}_{\left(CR\right)}=-1617.5+0.336\cdot ES+15.236\cdot ET+45.254\cdot LT-1.736\cdot PT-0.003\cdot {ES}^{2}-0.035\cdot {ET}^{2}\\ +\;0.016\cdot {PT}^{2}+0.002\cdot ES\cdot ET-0.674\cdot ES\cdot LT-0.005\cdot ES\cdot PT \end{array}$$

(2) $$\begin{array}{c} {\hat{Y}}_{\left(TS\right)}=-54.794-0.208\cdot ES+0.401\cdot ET+750.127\cdot LT-0.084\cdot PT-1011.512\cdot LT^{2}+0.880\cdot ES\cdot LT\\ \;\;\;\;-\;1.653\cdot ET\cdot LT \end{array}$$

### 2.2. Fabrication of Scaffolds, Tensile Strength, and Module of Elasticity Testing

**Figure 3:**
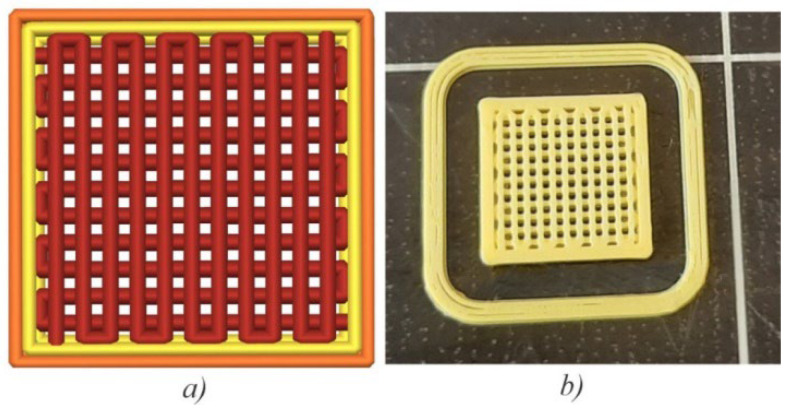
Scaffold prepared for 3D printing in Prusa Slicer (**a**), image of a 3D printed scaffold (**b**).

Both types of scaffolds used for cell seeding were printed on a 3D printer, Prusa i3 MK2S FFF (*Czech Republic*), with a steel 0.4 mm nozzle. A brand new, commercially available, yellow 1.75 mm PLA filament from 3D-Fuel (USA) was used as-is and did not undergo any previous treatment prior to extrusion. The main processing parameters and their levels are given in Table 1. Regardless of the crystallinity level, square scaffolds (10 × 10 × 1 mm) were printed using alternate (0/90°) raster orientation and 55% infill (Figure 3), which provided the required matrix porosity (Figure 3a,b) and were also reported in some recent studies featuring 3D-printed PLA scaffolds [26,27].

Tensile strength and elasticity modulus tests were performed in compliance with the ISO 527- 2: 2012 specification, on a universal tester, *Shimadzu EZ-LX*, using 50 mm/min and 1 mm/min crosshead speed, respectively, at room temperature (24 °C).

### 2.3. Differential Scanning Calorimetry Analysis

Differential scanning calorimetry (DSC) analysis was performed with TG/DSC 111 (SETARAM Instrumentation, Caluire, France), in a helium atmosphere with a total flow of 40 mL/min. DSC curves were recorded in the range of 25 to 220 °C for all the samples with a heating rate of 10 °C/min. Crystallinity was calculated based on Equation (3) [28]:

(3) $$X_{c}=\frac{{∆H}_{m}-{∆H}_{cc}-{∆H}_{r}}{{∆H}_{f}}$$

where ΔH_m_, ΔH_cc_, and ΔH_r_ represent the enthalpies of melting, cold crystallization, and reordering of polymer chains, respectively. The heat of fusion (ΔH_f_), corresponding to 100 percent melting enthalpy of crystalline PLA, was adopted as 93 J/g [28]. Material samples were taken from the central rectangular zone on the cross section, which was approximately equally distanced from the lower and upper surfaces.

### 2.4. Surface Characterization

Porosity examination and energy dispersive spectroscopy (EDS) mapping of the scaffolds were performed on a scanning electron microscope, SEM JEOL JSM 6460 LV. An accelerating voltage of 20 kV at ×50 and ×250 magnification was used for all images in the backscatter mode (BEI).

Microsurface topography was analyzed using a T2000 stylus profilometer (Hommelwerke, Germany). Profiles were acquired at a 4.8 mm cut-off length, obtaining a resolution of 1.2 µm in the X- and 0.1 µm in the Z-direction. Surface roughness parameters were determined according to the EN ISO 4287:2014 standard.

Nanosurface topography was evaluated on CP-II di (Veeco, Plainview, NY, USA). Atomic force microscopy (AFM) measurements were performed in contact mode using a symmetrically etched silicon-nitride probe. A scan rate of 0.5 Hz and a setpoint of 100 nN were employed. Images were acquired on 100 × 100 μm areas, which were subsequently used to acquire high-resolution images of 20 × 20 μm and 5 × 5 μm. All images have a lateral resolution of 256 × 256 pixels. Image analysis was performed using Spip 6.2.0 (Image Metrology, Lyngby, Denmark) image processing software, which was also used for the calculation of surface roughness parameters.

The static contact angles for the LLC and HLC scaffolds were determined using a sessile drop method with a drop shape analyzer by Krüss DSA25E (Hamburg, Germany). Droplets (1.3 μL) of demineralized water were deposited on the scaffold plate surface and the initial contact angle (θci) was measured after 1s at room temperature. A total of six samples (three of each type) were used and measurements were repeated five times for the mean and standard deviation calculation.

### 2.5. Cell Viability Tests and Biochemical Analyses

Human mesenchymal stromal cells (hMSC) were isolated from the bone marrow of healthy donors after informed consent was provided by Medical Clinic I of the Dresden University Hospital with the approval of the ethics commission of TU Dresden for in vitro experiments. The cells were expanded in a monolayer culture at 37 °C and 5% CO_2_ in a cell culture medium consisting of α modification of Minimum Essential Medium (α-MEM) (Dulbecco’s Modified Eagle’s Medium, Gibco, Thermo Fisher, Waltham, MA, USA) supplemented with 15% fetal calf serum (FCS), (Corning) and 1% P/S (100 U·mL^−1^ penicillin and 100 μg·mL^−1^ streptomycin, all from Biochrom, Berlin, Germany). Osteogenic cell differentiation was induced by the addition of 10^−8^ M dexamethasone, 0.05 × 10^−3^ M ascorbic acid 2-phosphate, and 10 × 10^−3^ M β-glycerophosphate to the cell culture medium. The cell culture medium was changed twice a week. When the cells reached the confluence at the fifth passage, they were trypsinized and 5 × 10^4^ cells were seeded onto PLA scaffolds. Seeded scaffolds were cultured under the same above-mentioned cell culture conditions. The experimental medium contained 9% fetal calf serum. A live/dead cell viability assay was performed by incubating cell-seeded scaffolds for 30 min with 2 mM Calcium AM and 4 mM Ethidium homodimer-1 in the cell culture medium. Subsequent imaging was performed on a fluorescence microscope Biorevo BZ-900 (Keyence, Neu-Isenburg, Germany) at magnifications of ×2 and ×10. For the biochemical assays, the cell-seeded scaffolds were frozen and thawed, and lysis of the cells was performed with a 1% Triton X-100 in PBS. QuantiFluor dsDNA system (Promega, Madison, WI, USA) was used for DNA quantification, while the measurements were performed on a microplate reader Infinite 200 PRO (Tecan, Switzerland) with excitations at 485 nm and emission at 535 nm. ALP tests were conducted using a substrate solution that contained 1 mg·mL–1 4-nitrophenylphosphate in 0.1 M diethanolamine, 0.1% Triton X-100, 1 mM MgCl2, pH 9.8 (all from Sigma-Aldrich, St. Louis, MI, USA). The enzymatic reaction was stopped with 1 M NaOH, while the absorbance was measured at 405 nm on the microplate reader. A calibration line was established using different concentrations of 4-nitrophenol. LDH activity was analyzed using CytoTox 96^®^ Nonradioactive Cytotoxicity Assay (Promega, Madison, WI, USA). Kinetics measurement at 490 nm absorbance was performed on a microplate reader Infinite 200 PRO (Tecan, Switzerland).

## 3. Results and Discussion

### 3.1. Surface Characterization

**Figure 4:**
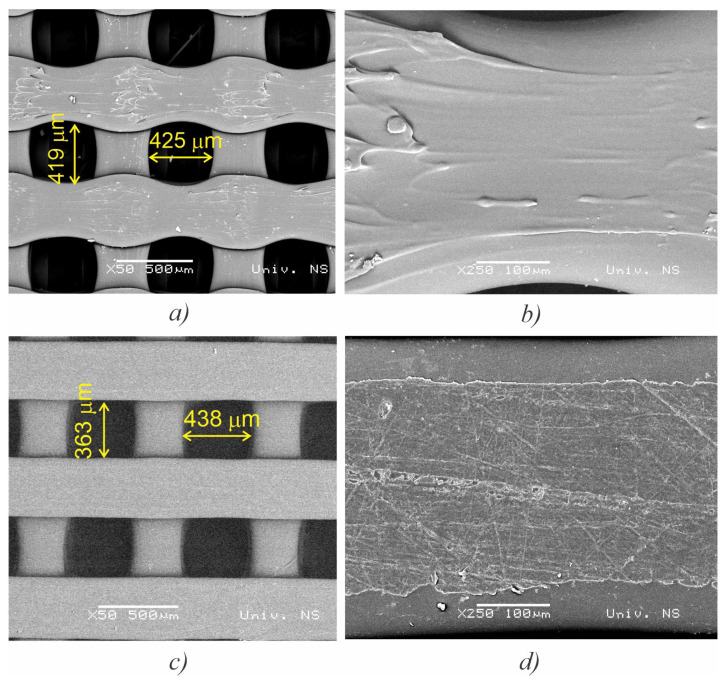
SEM micrographs showing grid morphology, pore size, and surface topography for the LLC (**a**,**b**), and HLC scaffolds (**c**,**d**).

Bearing in mind that 3D scaffold porosity and pore structure play a significant role in terms of cell penetration, distribution, vascularization, and mechanical forces [25,29,30], the selected fabricated specimens were examined by SEM for pore dimensions. Both scaffold types had pore sizes within the required range for human mesenchymal cells, i.e., 350–500 μm (Figure 4a,b). The scaffolds with a higher level of crystallinity (HLC) exhibited more geometric regularity of the extruded paths (Figure 4c) than their lower crystallinity (LLC) counterparts (Figure 4a). However, even low magnification reveals more variety in the surface topography of HLC specimens (Figure 4d) compared to the LLC ones (Figure 4b).

**Figure 5:**
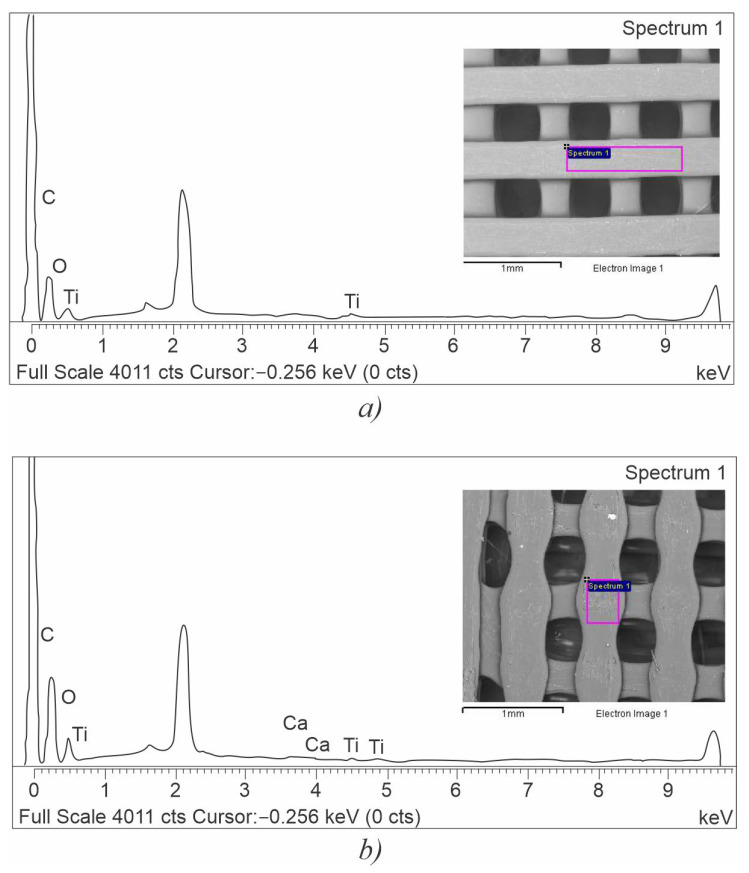
EDS elemental composition values [Wt.%] for (**a**) LLC and (**b**) HLC scaffolds.

Elemental composition values are shown in Table 2, while the graphic representation of EDS analysis is presented in Figure 5.

As shown in Table 2, there were no significant differences in EDS mappings of the two types of scaffolds. Both samples exhibited small Ti peaks which are associated with titanium oxide-based colorants present in the composition of a wide range of pigments used in filament manufacturing. Similar peaks have been previously reported for the plain 3D printed PLA by [31] and attributed to the TiO_2_ present in the white pigment. As shown in Table 2, the presence of Ca is reported for the LLC sample and can be attributed to calcium carbonate, also a common additive in the processing of polymers. It has been indicated in previous studies [8] that the presence of inorganic additives or fillers, as well as the chemical and structural changes produced by the FFF extrusion, can significantly impact surface reactivity, functionalization, and association with cells and organisms.

However, in this experiment, no significant differences were observed between the two types of scaffolds which, in turn, could lead to significant differences in cell response.

### 3.2. Crystallinity Content, Tensile Strength, and Module of Elasticity

**Figure 6:**
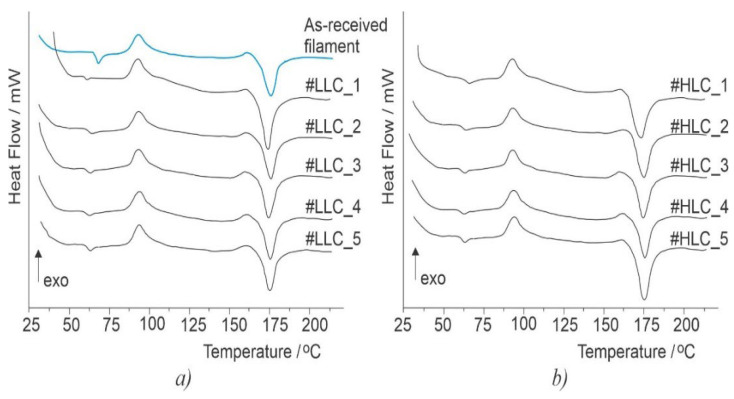
DSC curves for the LLC (**a**) and HLC samples (**b**).

Before engaging with the main part of the experiment, the validity of crystallinity content predicted by the empirical model given in Equation (1) was checked. Two sets of five samples were printed using the LLC and HLC settings, and their crystallinity content was analyzed by the DSC. The results for LLC and HLC scaffolds are presented in Table 3 and Table 4, respectively, while the DSC thermograms are shown in Figure 6. Both crystallinity means (Figure 1 and Figure 2) predicted by Equation (1) lie within the experimentally obtained 95% confidence intervals (CI), LLC 95%CI = [6.81, 12.34] and HLC 95%CI = [14.44, 19.44]. These results show that the empirical model is valid and they are also in line with the recent reports that higher build plate temperature [32] and lower extrusion speed [33] promote crystallization phenomena.

Similar to crystallinity content, tensile strength was tested to verify the prediction model in Equation (2). The results are shown in Table 5 and Table 6. The model given in Equation (2) passed validation, considering that both predicted tensile strength values (Figure 1 and Figure 2) were within the experimentally obtained 95% confidence intervals, LLC 95%CI = [61.63, 65.30] and HLC 95%CI = [61.87, 66.13].

The module of elasticity results are given in Table 7. There was no significant difference in module of elasticity t(4) = 1.16, *p* = 0.308 between the LLC (M = 1.448, SD = 0.140) and HLC samples (M = 1.545, SD = 0.08). It has been well established that different human organs exhibit different matrix stiffnesses. Thus, according to their lineage, the cells prefer environments with specific stiffness, which must also be mimicked by the scaffolds in order to promote their differentiation.

The results show that both types of polymer matrices feature stiffness within the 1–10 GPa range which favors osteoblast growth and differentiation [34]. Therefore, in this study, stiffness is an unlikely contributor to the observed difference in cell response.

### 3.3. Micro- and Nanosurface Topography Analysis Results

In order to systematically examine the surface topography of the scaffolds, micro- and nanosurface topographies were characterized using the amplitude, spatial, and hybrid roughness parameters.

#### 3.3.1. Microsurface Topography

**Figure 7:**
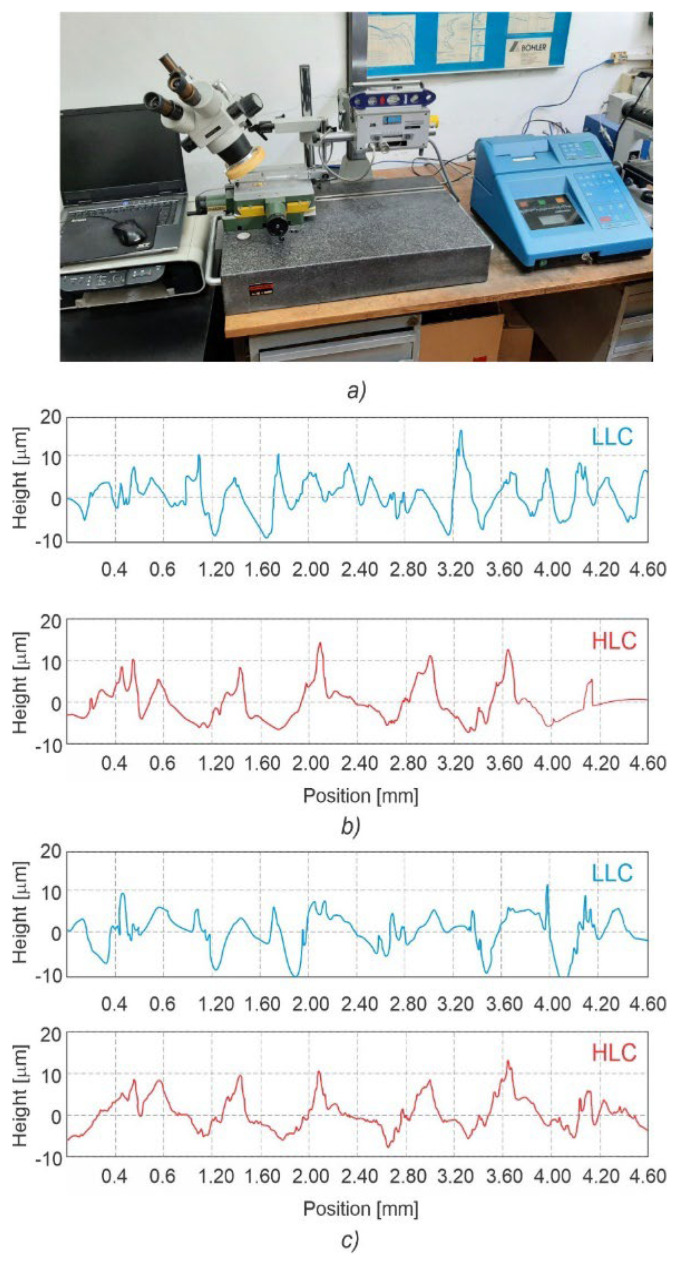
Positioning of the stereo microscope during measurements (**a**), and the two selected pairs of LLC and HLC scaffold plate profiles (**b**,**c**), which exhibit a visible difference in the width of mean profile elements (Rsm).

To allow precise stylus positioning above the extruded paths during microsurface topography measurements, a stereo microscope was mounted as shown in Figure 7a. The only statistically significant difference in the microsurface topography parameters between the LLC and HLC scaffolds was noted in the spatial domain, namely in the mean width of profile elements, Rsm (Table 8). The statistically significant difference between the two averages (t(8) = −2.74, *p* = 0.025), indicates a difference in horizontal profile periodicity, which is also illustrated in Figure 7b,c.

#### 3.3.2. Nanosurface Topography

**Figure 8:**
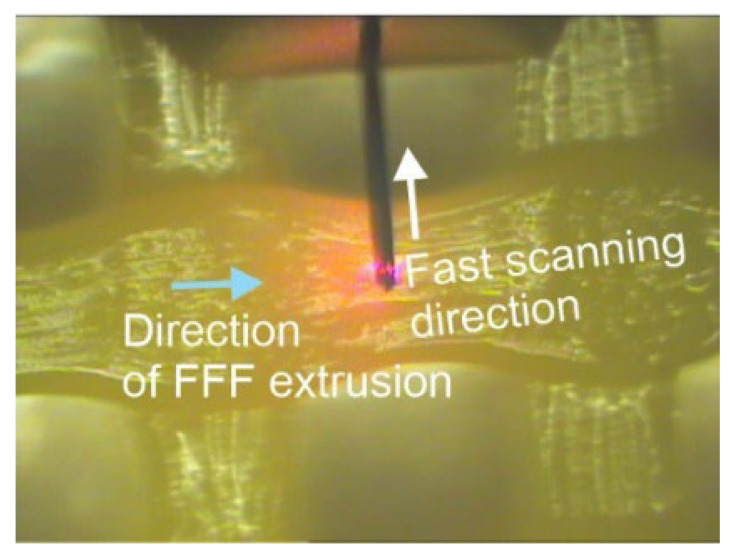
Fast scanning direction relative to the direction of FFF extrusion, as used for obtaining the AFM scans shown in Figure 9 and Figure 10.

**Figure 9:**
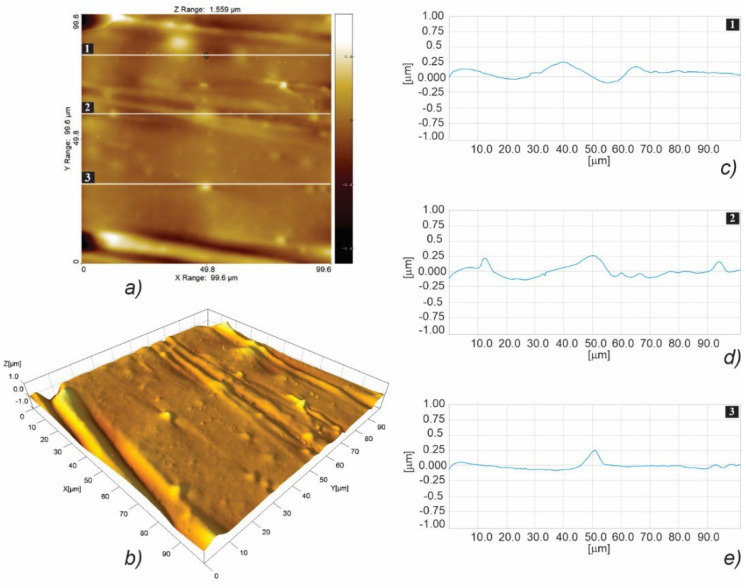
AFM topographic images obtained for the LLC scaffold over 100 × 100 µm area (**a**,**b**), and the corresponding profiles taken at locations marked with 1 (**c**), 2 (**d**), and 3 (**e**).

**Figure 10:**
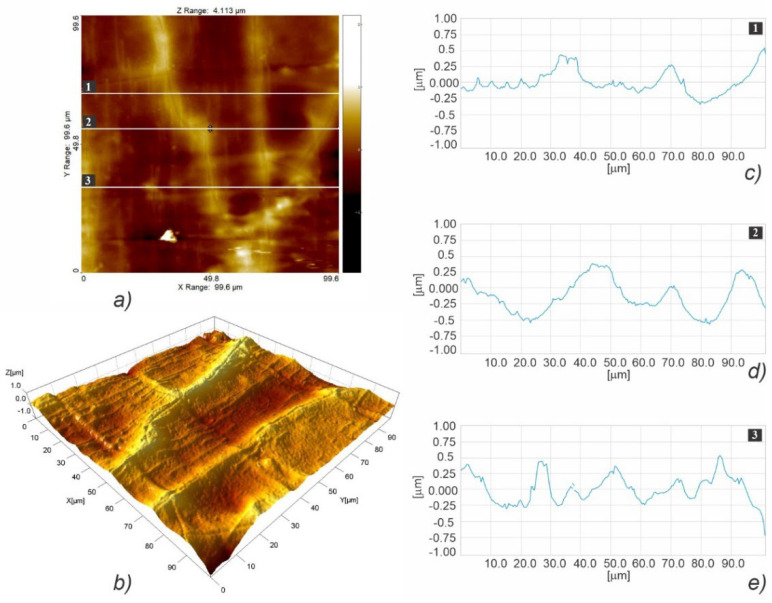
AFM topographic images obtained for the HLC scaffold over 100 × 100 µm area (**a**,**b**), and the corresponding profiles taken at locations marked with 1 (**c**), 2 (**d**), and 3 (**e**).

**Figure 11:**
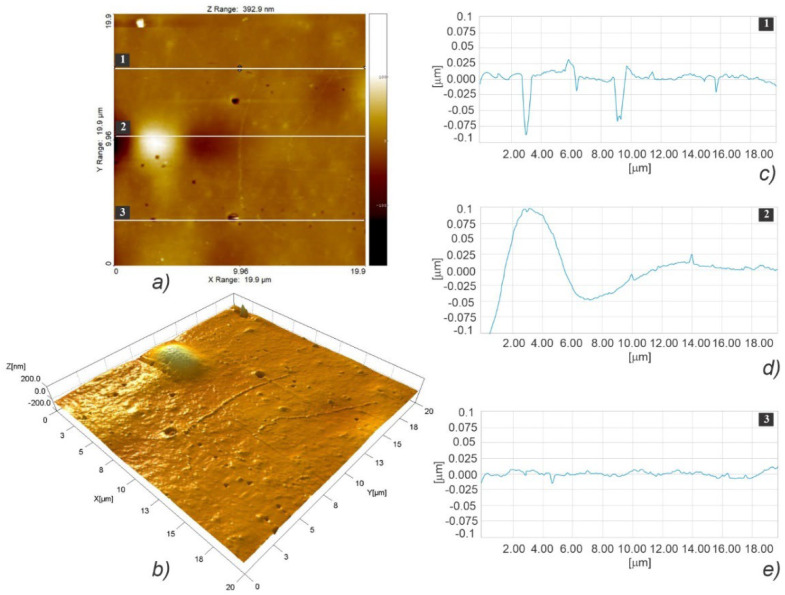
AFM topographic images obtained for the LLC scaffold over a 20 × 20 µm area (**a**,**b**), and the corresponding profiles taken at locations marked with 1 (**c**), 2 (**d**), and 3 (**e**).

**Figure 12:**
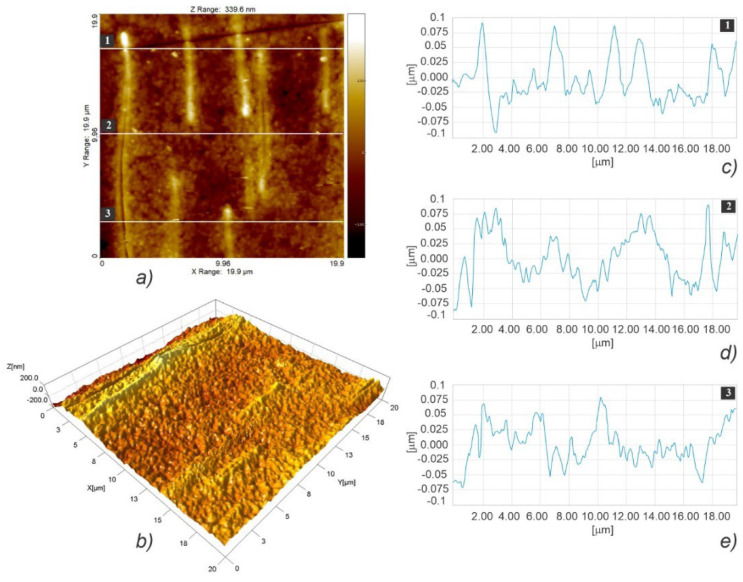
AFM topographic images obtained for the HLC scaffold over 20 × 20 µm area (**a**,**b**), and the corresponding profiles taken at locations marked with 1 (**c**), 2 (**d**), and 3 (**e**).

**Figure 13:**
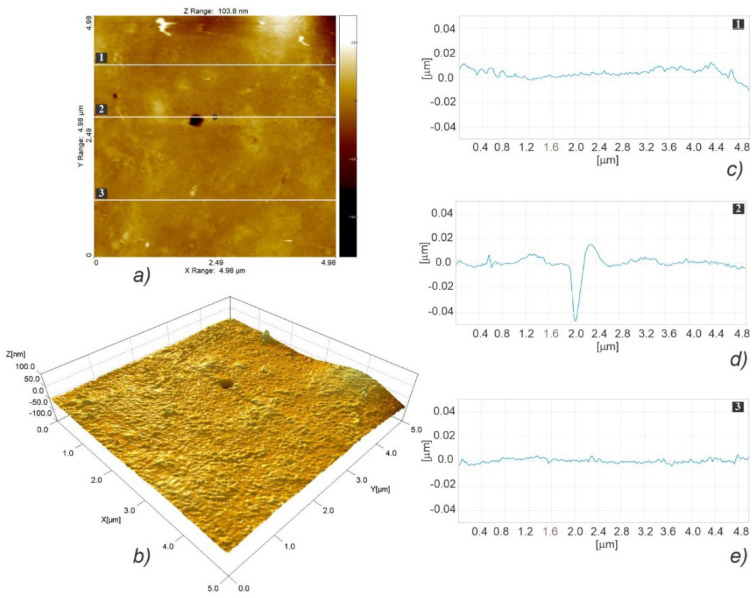
AFM topographic images obtained for the LLC scaffold over 5 × 5 µm area (**a**,**b**), and the corresponding profiles taken at locations marked with 1 (**c**), 2 (**d**), and 3 (**e**).

**Figure 14:**
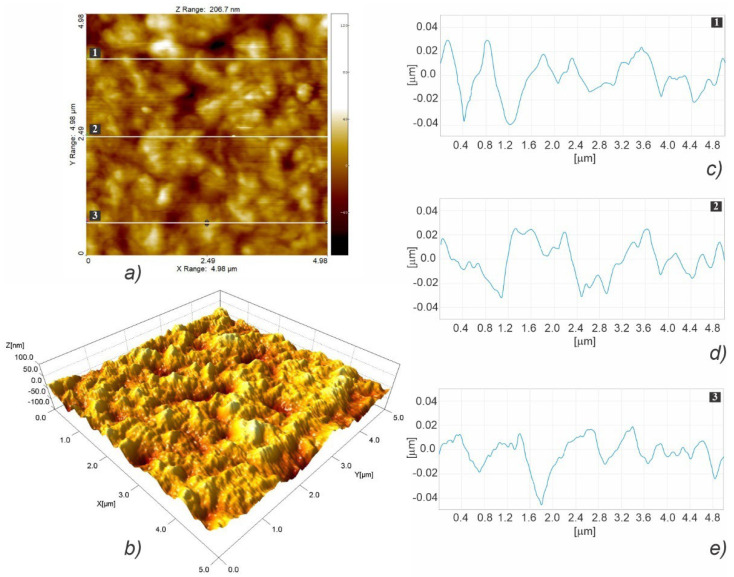
AFM topographic images obtained for the HLC scaffold over 5 × 5 µm area (**a**,**b**), and the corresponding profiles taken at locations marked with 1 (**c**), 2 (**d**), and 3 (**e**).

Figure 8 illustrates the fast scanning direction relative to that of material extrusion, while the AFM scan images and profile curves for LLC and HLC scaffolds are shown in Figure 9, Figure 10, Figure 11, Figure 12, Figure 13 and Figure 14. Visual examination of 3D scan images (Figure 9b, Figure 10b, Figure 11b, Figure 12b, Figure 13b and Figure 14b) reveals that LLC and HLC topographies are dominated by different micro- and nanofeatures. The LLC scaffolds have smooth grooves and protrusions that stretch in the FFF extrusion direction. Contrastingly, the HLC scaffolds are characterized by micrometer-scale bulged oblong protrusions of different heights that stretch perpendicularly to the FFF extrusion direction. The differences are also well illustrated by the profile curves extracted from the scan images taken at three locations for each scan (Figure 9c–e, Figure 10c–e, Figure 11c–e, Figure 12c–e, Figure 13c–e and Figure 14c–e). Further insight is enabled by a detailed examination of topography measurement results (Table 9, Table 10 and Table 11). The reported results are expressed as mean values with standard deviations. Surface roughness parameters were calculated for the measurements performed over the areas of 100 × 100 mm.

The root-mean-square roughness (Sq) (Table 9) represents the standard deviation of the distribution of surface heights. Although the *p* value obtained by the t-test is marginally above the 0.05 threshold (t(4) = 2.65, *p* = 0.057), the nanoscale features of the two types of scaffolds are distinguishable, since the Sq obtained for HLC scaffolds are almost two times larger. As regards the surface skewness (Table 9), Ssk measurements did not show any statistically significant difference between the two types of scaffolds (t(4) = −0.02, *p* = 0.984). Both HLC and LLC scaffolds exhibit positive skewness, which indicates that they are peak dominated since their height distribution is skewed above the mean plane. Due to the large standard deviation, which characterizes surface kurtosis (Sku) obtained for LLC scaffolds, no statistically significant difference was observed in the Sku means for the two types of scaffolds (t(2) = −2.63, *p* = 0.119). However, it is obvious that, in the case of the LLC scaffolds, a Sku over nine indicates a leptokurtic profile where a larger proportion of the deviation from the mean can be attributed to a few particularly high and/or particularly low height readings. The fastest decay autocorrelation length (Scl37) (Table 10) was a length parameter that describes the character of the areal autocorrelation function (AACF) and is the length over which the function decays to a threshold value. For an isotropic surface, the AACF decays identically fast in all directions. In the case of anisotropic surfaces, the AACF decays rapidly along the cross-lay direction while the decay is slow along the lay direction. Although at first look, the results might indicate that the Scl37 for the HLC scaffolds is greater than that of the LLC scaffolds, the test was insignificant (t(4) = 1.49, *p* = 0.210) suggesting that one cannot claim the difference in texture isotropies of the two types of scaffold surface patterns. The texture aspect ratio of the surface (Str37) (Table 10) is used to identify the topographic texture pattern together with the fastest decay autocorrelation length. Calculated based on the AACF, this parameter is defined as the ratio of the shortest decay distance to the longest decay distance at a normalized threshold of 0.37, and its value ranges between zero and one. While larger values, above 0.5, suggest a uniform texture in different directions, smaller values, of 0.3 and lower, indicate strong anisotropy and texture in one direction. In the case of our samples, a statistically significant difference was established (t(4) = −4.56, *p*.01) between the two averages, suggesting that the texture nonuniformity is higher in the HLC scaffolds, which exhibit Str37 closer to 0.3 value. The density of summits (Sds) (Table 10) denotes the number of summits per unit sampling area, where the points above the mean plane of a specified area that have the largest value compared to other data points are considered summits. Compared to their LLC counterparts, the HLC scaffolds exhibited a significantly larger number of summits per unit sampling area (t(4) = 3.57, *p* = 0.023). For the assessment of the root mean square slope of the surface (Sdq) (Table 11), the slopes are calculated between every adjacent data point in the x and y directions. Thus, surfaces that feature height steps or sharp spikes have greater values of this parameter. In our case, the patterns of the HLC scaffolds exhibited significantly greater slope values than their LLC counterparts (t(4) = 5.51, *p* = 0.005), suggesting that the LLCs feature smoother, i.e., wider spaced, texture in surface height over the same spacing. Over the sampling area, the developed surface area ratio (Sdr) (Table 11) is calculated from the ratio of the rough surface to a flat surface. Theoretically, for an ideally flat surface, the Sdr would equal zero, while this value would increase with the increased local slope variation. The Sdr value obtained for the HLC scaffolds is significantly higher than that of the LLC scaffolds (t(4) = 4.59, *p* = 0.01), which leads to the conclusion that HLC scaffolds exhibit a more prominent height contrast on the surface. This is clearly visible when comparing images in Figure 13b and Figure 14b. Representing the average of the principal curvatures of the summits defined by the AACF length, the mean summit curvature (Ssc) (Table 11) is considered to be a measure of the general shape and dimension of the summits. The results show that the value obtained for the HLC scaffolds is significantly higher (t(4) = 6.35, *p* = 0.003) than that of the minimum crystallinity (LLC) scaffolds, suggesting a generally larger effective diameter of the summits on the HLC scaffolds.

Overall, the results of nanotopography show that there is no significant difference in texture isotropy between the two types of scaffolds. The HLC scaffolds exhibited a larger texture nonuniformity and height contrast on the surface, as well as a larger number and effective diameter of summits per sampling area. In contrast, compared to their HLC counterparts, LLC scaffolds had a smoother, wider-spaced texture. Regarding the cell response results shown in Section 3.5 which are in favor of the HLC scaffolds, the observed differences in nanosurface topography indicate that increased surface roughness at the nanoscale promotes cell adhesion and proliferation.

### 3.4. Contact Angle Measurement

**Figure 15:**
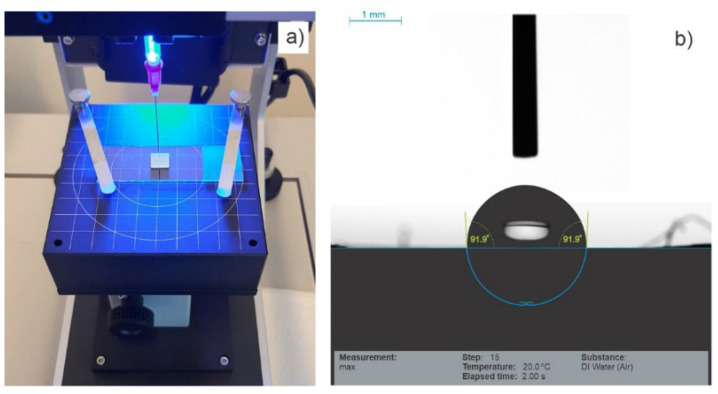
Contact angle measurements: (**a**) scaffold positioned for measurement on the work table, (**b**) an image obtained for an HLC scaffold.

In order to determine if the level of crystallinity affects the contact angles of the two types of scaffold surfaces, contact angles were measured as shown in Figure 15 and Table 12. No significant difference, t(4) = 0.24, *p* = 0.819, in static angles was found between the LLC (M = 94.32, SD = 2.56) and HLC (M = 94.91, SD = 3.36) types of scaffolds, as both types exhibited slight hydrophobicity. Interestingly, the difference in various aspects of their nanosurface topographies, discussed in Section 3.3.2, did not impact their wettability. Although reports in the literature include experiments with various cell types on different polymer surfaces of various wettability, we are still far from fully understanding the mechanisms which are decisive for cell adhesion, proliferation, and differentiation. The main reason is the sheer complexity of this problem, which is affected not only by surface wettability but also by the interaction of numerous factors such as the polymer surface roughness, chemical components of the polymer surface, etc.

### 3.5. Biological Characterization

**Figure 16:**
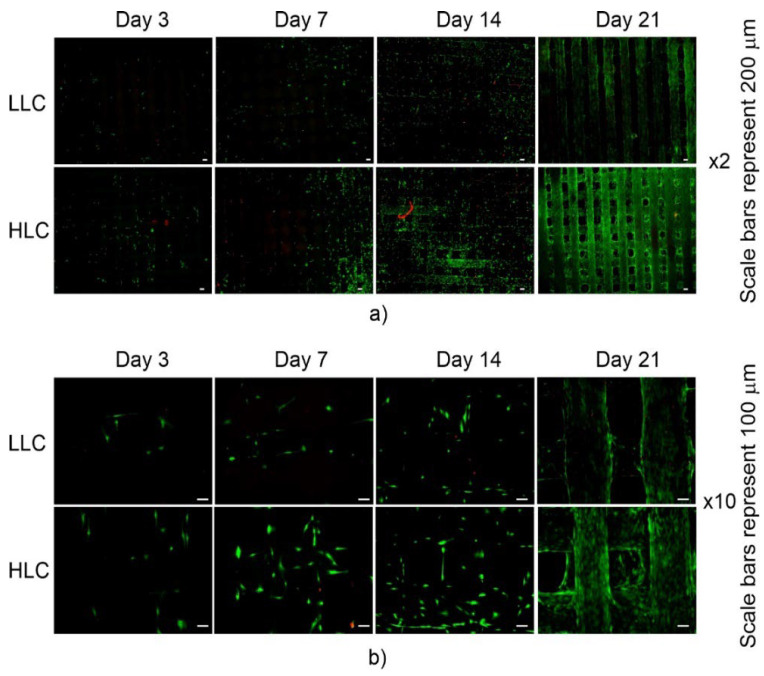
Microscopic images after live-dead assay using fluorescence staining of cells seeded on top of LLC and HLC scaffolds over 21 days at (**a**) lower (×2) and (**b**) higher (×10) magnification.

**Figure 17:**
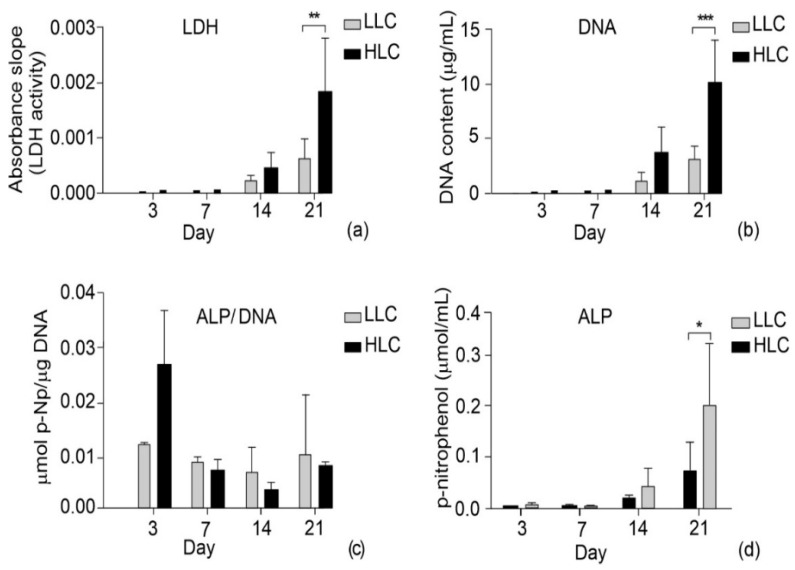
Viability (LDH activity) (**a**), quantification of DNA (**b**), sample-specific differentiation (ALP activity/DNA) (**c**), and differentiation (ALP activity) (**d**) over 21 days of cells seeded on top of LLC and HLC scaffolds, *p* < 0.05(*), *p* < 0.01(**), *p* < 0.001(***).

Previous studies have demonstrated that different micro- and nanotopographies affect human cell behavior [35,36]. Adhesion, spreading, growth, and differentiation of primary human osteoprogenitor cells can be controlled by different nanotopographies [37]. In addition to topography, the crystallinity of biomaterials can influence human cell responses. By changing the crystallinity of polycaprolactone (PCL), scaffold cell adhesion, proliferation, and gene expression were significantly affected [38]. With this short discussion in mind, a biological characterization of the scaffolds was conducted in vitro by testing the viability, proliferation, and differentiation of seeded cells. Cell viability was tested through live-dead assays and measurement of lactate dehydrogenase (LDH) activity. Shown in Figure 16 are the two-color assays which illustrate the cell viability tests, labeling the live and dead hMSC cells in green and red, respectively. The results pertain to the 3rd, 7th, 14th, and 21st days of the experiment. The green signal indicates the cytoplasm of the metabolically active (alive) cells, while the nuclei of the dying and dead cells are indicated by the red signal. The fluorescent microscopic images were taken under lower (2×) and higher (10×) magnification. Lower magnification revealed the overall quality of cell spread over the entire scaffold (Figure 16a), while the shape of cells was better observed under higher magnification (Figure 16b) where the viable cells were well spread and assumed elongated shapes. Comparison between the microscopic images in Figure 12b reveals that the difference between the LLC and HLC scaffolds became more apparent as the experiment progressed. This finding is in line with the results from [15] where the proliferation of human osteoblast cells on PLLA substrates was promoted by higher crystallinity. LDH is a marker of cell viability, corresponding to metabolic activity, and in normal conditions increases with the number of proliferating cells over time. The statistically significant difference in LDH activity of the cells seeded on LLC and HLC scaffolds was only observed on Day 21 (Figure 17a).

DNA content was measured as a precise way to assess cell proliferation in cell populations on both types of plates. The DNA quantification shows a similar trend to that of the LDH, suggesting that the cells were metabolically active. Since the DNA quantity depends on the total number of cells, this result suggests that most of the cells on the scaffold were metabolically active. As shown in Figure 17b, a statistically significant difference in DNA quantity was only reached at the final stage of the experiment, i.e., on day 21.

In terms of early osteoblastic differentiation, the highest activity was observed on day three (Figure 17c), where the specific ALP activity was significantly in favor of the HLC scaffolds. However, this result is not surprising, considering the very low content of measured DNA on these samples. Since proliferating cells do not differentiate, the ALP and DNA values are low at later time points where higher proliferation was observed (Figure 17c). Looking at the results of the actual measured ALP activity (Figure 17d), higher osteogenic differentiation was observed at later time points, achieving statistical significance on day 21. Similar results were observed in another study with PLA, where the increase of material surface topography induced higher differentiation of mesenchymal stem cells towards osteoblastic lineage [38]. In perspective, it would be interesting to extend biological experiments with different cell donors, since it has been shown that a patient’s age can affect cellular response to the micro- and nanotopography of materials [39]. With that in mind, it is possible that different health conditions of donors can further affect cell response. All this suggests that biomaterial surface topography could be controlled in order to customize it to a patient’s health condition, thus contributing to personalized implant therapies. Finally, more detailed information on cell differentiation could be revealed by later markers, such as osteopontin or bone sialoprotein [40].

## 4. Conclusions

In this study, an empirical model featuring four FFF processing parameters was used to regulate the level of in-process crystallinity and tensile strength of scaffolds. Two different processing regimes were used to fabricate scaffolds with low and high levels of crystallinity while keeping their tensile strengths as high as possible. The module of elasticity, contact angle, and micro- and nanosurface topography were also examined in detail. The results have shown that the two FFF processing regimes yielded microsurface topographies that were only significantly different in the horizontal profile periodicity. Contrastingly, the two types of scaffolds exhibited entirely different nanotopographies, which, in turn, significantly impacted their cell response. In comparison with their low-level crystallinity counterparts, high-level crystallinity scaffolds featured higher nonuniformity and a larger number of summits per sampling area. In contrast, low crystallinity yielded smoother and wider spread nanotexture. As the result, high-level crystallinity scaffolds performed significantly better in terms of cell response. Presently, FFF technology cannot match the precise control over the created nanoenvironment offered by the advanced and intricate methods for the generation of synthetic, regularly shaped, and distributed polymer nanostructures. However, the most striking observation from this study was the fact that the adequate selection of FFF printing parameters can produce significantly different nanotopographies. Moreover, our in vitro study has demonstrated that FFF extrusion already has the potential to be a fast, easy, and cost-effective way to fabricate scaffolds with a favorable cell response, which requires no postprocessing. Should this approach receive wider attention from researchers in the near future, it could soon support favorable biological responses in vivo.

## Figures and Tables

**Table 1:** Main processing parameters used for the FFF extrusion of LLC and HLC scaffolds.

Scaffold Plate Type	FFF Processing Parameter			
	Extrusion Speed (mm/s)	Extruder Temperature (°C)	Layer Thickness (mm)	Build Plate Temperature (°C)
Low-level cryst. (LLC)	90	230	0.2	60
High-level cryst. (HLC)	30	220	0.2	80

**Table 2:** EDS elemental composition values [Wt.%] of LLC and HLC scaffolds.

Scaffold Plate Type	C [wt.%]	O [wt.%]	Ti [wt.%]	Ca [wt.%]
LLC	62.02 ± 1.28	37.05 ± 1.29	0.63 ± 0.15	0.30 ± 0.11
HLC	62.62 ± 2.00	35.73 ± 2.02	1.65 ± 0.32	-

**Table 3:** Crystallinity contents reported for the five LLC scaffolds.

LLCSpecimen Number	Crystallization Enthalpy ΔH_c_ (J/g)	Recrystallization Enthalpy ΔH_r_ (J/g)	Melting Enthalpy ΔH_m_ (J/g)	Crystallinity X_c_ (%)
#1	29.73	4.10	42.21	9.01
#2	27.78	3.47	40.21	9.63
#3	22.79	3.89	38.58	12.79
#4	25.55	5.49	37.14	6.56
#5	26.24	4.58	40.01	9.90

**Table 4:** Crystallinity contents reported for the five HLC scaffolds.

HLC Specimen Number	Crystallization Enthalpy ΔH_c_ (J/g)	Recrystallization Enthalpy ΔH_r_ (J/g)	Melting Enthalpy ΔH_m_ (J/g)	Crystallinity X_c_ (%)
#1	18.78	4.40	36.24	14.04
#2	21.07	4.17	41.76	17.77
#3	18.255	3.29	37.054	16.68
#4	20.56	3.98	40.016	16.64
#5	20.17	3.67	42.05	19.58

**Table 5:** Results of experimental verification for tensile strength, obtained with parameter settings from dual-response optimization for LLC scaffolds (Figure 1).

**Force** **(N)**	511.8	497.4	505.8	511.8	480.7	535.7	513.0	524.9
**Tensile Stress** **(MPa)**	63.97	62.17	63.22	63.97	60.08	66.96	64.12	65.61

**Table 6:** Results of experimental verification for tensile strength, obtained with parameter settings from dual-response optimization for HLC scaffolds (Figure 2).

**Force** **(N)**	502.1	480.5	525.3	500.8	510.7	550.2	507.8	518.6
**Tensile Stress** **(MPa)**	62.76	60.10	65.66	62.6	63.84	68.77	63.47	64.82

**Table 7:** Values of elasticity modulus obtained for LLC and HLC specimens.

Specimen No.	Yield Tensile Force (0.20%) (N)	Yield Stress (0.20%) (MPa)	Yield Strain (0.20%)	Young’s Modulus (GPa)
#1 LLC	1027.4	26.195	1.981	1.322
#2 LLC	1132.9	28.695	2.052	1.398
#3 LLC	1292.5	32.737	2.054	1.593
#4 HLC	1142.0	28.972	1.802	1.607
#5 HLC	1191.3	29.964	1.911	1.567
#6 HLC	1123.1	28.278	1.937	1.460

**Table 8:** Amplitude (Rq, Rsk, and Rku), spacing (Rsm), and hybrid (Rdq) roughness parameters of microsurface topography.

Scaffold Plate Type	Root Mean Square (R_q_ ± SD) [μm] (*n* = 6)	Surface Skewness (R_sk_ ± SD) (*n* = 6)	Surface Kurtosis (R_ku_ ± SD) (*n* = 6)	Mean Width (R_sm_ ± SD) [μm] (*n* = 6)	Root Mean Square Slope (R_dq_ ± SD) (*n* = 6)
LLC	3.507 ± 0.278	0.677 ± 0.596	3.960 ± 0.573	364.25 ± 64.39	0.146 ± 0.02
HLC	3.646 ± 0.785	0.967 ± 0.210	3.768 ± 0.746	510.95 ± 100.79	0.145 ± 0.037

**Table 9:** Amplitude roughness parameters of nanosurface topography: Root-mean-square deviation of the surface (Sq), skewness of surface height distribution (Ssk), and kurtosis of surface height distribution (Sku).

Scaffold Type	Root Mean Square (Sq ± SD) [nm] (*n* = 3)	Surface Skewness (Ssk ± SD) (*n* = 3)	Surface Kurtosis (Sku ± SD) (*n* = 3)
LLC	115.13 ± 43.59	0.453 ± 1.017	9.048 ± 3.765
HLC	225.30 ± 57.35	0.465 ± 0.055	3.281 ± 0.513

**Table 10:** Spacing roughness parameters of nanosurface topography: Fastest decay autocorrelation length (Scl37), texture aspect ratio (Str37), and density of summits (Sds).

Scaffold Type	Fastest Decay Autocorrelation Length (S_cl37_ ± SD) [nm] (*n* = 3)	Texture Aspect Ratio (S_tr37_ ± SD) (*n* = 3)	Density of Summits (S_ds_ ± SD) [1/μm^2^] (*n* = 3)
LLC	4687.5 ± 2067	0.484 ± 0.089	0.124 ± 0.036
HLC	6510.4 ± 451	0.350 ± 0.099	0.257 ± 0.053

**Table 11:** Hybrid roughness parameters of nanosurface topography, Root-Mean-Square slope of the surface (Sdq), developed surface area ratio (Sdr), and arithmetic mean summit curvature of the surface (Ssc).

Scaffold Type	RMS Surf. Slope (S_dq_ ± SD) (*n* = 3)	Developed Surf. Area Ratio (S_dr_ ± SD) (*n* = 3)	Arith. Mean Summit Curv. (S_sc_ ± SD) [1/nm] (*n* = 3)
LLC	0.0513 ± 0.007	0.133 ± 0.036	131 × 10^−6^ ± 41 × 10^−6^
HLC	0.106 ± 0.016	0.571 ± 0.161	348 × 10^−6^ ± 43 × 10^−6^

**Table 12:** Results of contact angle measurements for the LLC and HLC scaffolds.

Scaffold Type	Sample No.	Contact Angle (Sdq ± SD) [°] (*n* = 5)
LLC	#1	96.68 ± 0.658
	#2	91.60 ± 0.639
	#3	94.67 ± 0.321
HLC	#1	91.25 ± 0.557
	#2	97.84 ± 0.204
	#3	95.65 ± 0.464

## Data Availability

Data are available on request from the authors.
